# TCR mimic compounds for pHLA targeting with high potency modalities in oncology

**DOI:** 10.3389/fonc.2022.1027548

**Published:** 2022-10-21

**Authors:** Hans-Peter Gerber, Leonard G. Presta

**Affiliations:** ADC Research and Development Codeable Therapeutics, Palo Alto, CA, United States

**Keywords:** TCR mimic compounds, high potency modalities, protein binder scaffolds, targeted cancer therapy, cancer testis antigen, Antibody Drug Conjugates, CAR T, Bispecifics

## Abstract

pHLA complexes represent the largest class of cell surface markers on cancer cells, making them attractive for targeted cancer therapies. Adoptive cell therapies expressing TCRs that recognize tumor specific pHLAs take advantage of the unique selectivity and avidity of TCR: pHLA interactions. More recently, additional protein binding domains binding to pHLAs, known as TCR mimics (TCRm), were developed for tumor targeting of high potency therapeutic modalities, including bispecifics, ADCs, CAR T and -NK cells. TCRm compounds take advantage of the exquisite tumor specificity of certain pHLA targets, including cell lineage commitment markers and cancer testis antigens (CTAs). To achieve meaningful anti-tumor responses, it is critical that TCRm compounds integrate both, high target binding affinities and a high degree of target specificity. In this review, we describe the most advanced approaches to achieve both criteria, including affinity- and specificity engineering of TCRs, antibodies and alternative protein scaffolds. We also discuss the status of current TCRm based therapeutics developed in the clinic, key challenges, and emerging trends to improve treatment options for cancer patients treated with TCRm based therapeutics in Oncology.

## Introduction

**Figure 1:**
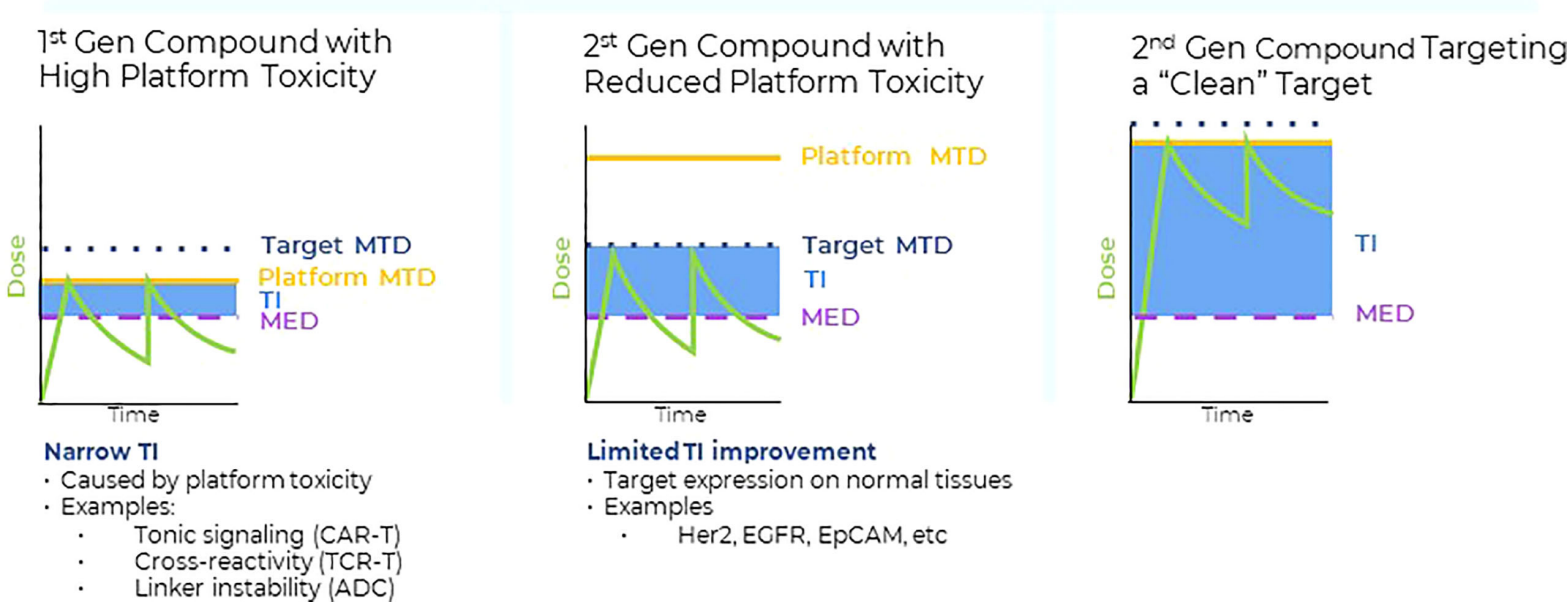
Left panel: Many first-generation high potency compounds developed in oncology displayed significant platform toxicities that were not target related. Such platform toxicities were either caused by linker instability (ADC), tonic signaling (CAR T), target cross-reactivities (TCRs) or immunogenicity (bispecifics). For many first generation, high potency modalities, the maximum tolerated dose levels (MTD) in the clinic were defined by the platform toxicity. The therapeutic index (TI) is a product of the differences between the minimum effective dose (MED) required to achieve anti-tumor activity and either the on target-, or platform MTD. Platform toxicities frequently limited the drug exposure of first-generation high potency compounds to levels that were close to the levels required for anti-tumor responses (i.e. narrow TIs). Middle panel: Protein engineering approaches were successfully applied to reduce platform related toxicities, enabling higher clinical dose levels and consequently, drug exposure levels with the purpose to increase the therapeutic index. However, despite the reduction in platform toxicities, the cell surface antigens expression on normal tissues posed a significant obstacle to increase overall drug exposure levels and anti-tumor activities of second-generation high potency compounds. Thus, despite the successful reduction of the initial platform toxicity, normal tissue expression of conventional cell surface antigens interfered with the full potential of high potency modalities, resulting in only moderate TI improvements. Right panel: Combining second generation, high potency modalities with reduced platform toxicity (middle panel) with targets that have higher tumor to normal tissue expression ratios, such as pHLA targets, provides a framework to fully leverage the progress made in platform engineering and to improve the TIs of high potency modalities. Some tumor specific, intracellular pHLA targets are not present on normal tissues (Tumor specific antigens (TSAs)), or are displayed at significantly lower levels (Tumor associated antigens, TAAs) (5). Targeting pHLA targets belonging to the TAA or TSA class with high affinity and specificity TCRm compounds may enable higher dose levels and improved anti-tumor effects in solid tumors.

When targeting conventional cell surface antigens with high potency modalities, including chimeric antigen receptor T-cells (CARTcells), bispecifics or antibody drug conjugates (ADCs), frequent onset of on-target, off-tumor toxicities were reported, caused by the expression of the target antigen on normal tissues. These circumstances limited the therapeutic exposure levels of most high potency compounds administered to solid tumor patients and consequently, their anti-tumor activities. The development of dose limiting toxicities on normal tissues significantly reduced the number of high potency compounds advancing to late-stage clinical trials in solid tumors [reviewed in (1–4)]. To overcome these limitations, it was proposed that targeting of the more tumor specific class of intracellular targets, which are presented on the cell surface in the form of short peptides bound by major histocompatibility (MHC) class I (MHC I) or MHC class II (MHC II) molecules, also known as human leukocyte antigens (HLAs) in humans. Targeting of a subset of peptide HLA (pHLA) targets, in particular the class of tumor specific intracellular targets, carries the potential to increase drug exposure levels and consequently, their therapeutic indexes which may ultimately translate into deeper and more durable anti-tumor responses [**Figure 1**, (6)]. However, this approach is contingent on the exquisite tumor specific expression of the pHLA target and minimal off-target, off-tumor cross reactivities of the compounds targeting them.

Recently, this therapeutic hypothesis was clinically validated by the unprecedented clinical response rates of uveal melanoma cancer patients treated with a CD3 bispecific targeting the pHLA target gp100, known as T cell receptor mimic (TCRm)-CD3 bispecific compounds. Gp100 represents a lineage commitment marker gene upregulated in melanoma cells of cancer patients, 7). The clinical benefit, in particular the improvement in the one-year overall survival (OS) and the progression free survival (PFS) endpoints, were significant compared to control therapy among previously untreated patients with metastatic uveal melanoma. Importantly, comparable improvements in survival have not yet been reported for CD3 bispecific compounds targeting conventional, cell surface antigens in solid tumors. The gp100-TCRm-CD3 bispecific compound is now approved for solid tumor targeting and represents a promising additional treatment option for patients. Furthermore, these data demonstrated that targeting of a low copy number pHLA target with a TCRm-CD3 bispecific can induce meaning full anti-tumor responses in solid tumors and indicate that pHLA targets carry the potential to replace conventional cell surface antigens in solid tumors, in particular when belonging to the class of lineage commitment- or cancer testis antigens (8).

## Differences between IgG- and TCR-mediated immune responses and impact on therapeutic engineering strategies in Oncology

During a productive immune response of the adaptive immune system, two different pathways are concomitantly engaged to induce immunity towards foreign pathogens or self-antigens. These pathways are known as cellular immune response, carried out by T cells and their T cell receptors (TCRs) and the humoral immune response, which is controlled by B cells and mediated by antibodies (Abs) produced by them. In general, cell surface antigens are recognized by Abs, whereas most intracellular targets are recognized by TCRs when presented as pHLA complexes. Two classes of T cells harboring distinct effector mechanisms can be differentiated based on the expression of either CD4 (helper T cells) or CD8 (killer T cells) co-receptors. CD4+ T cells recognize antigens in the context of MHC II and orchestrate the adaptive immune response by secretion of cytokines with pro-inflammatory, chemotactic or immunosuppressive properties. CD8+ T cells recognize antigens bound by MHC I complexes and carry out direct cytotoxic killing of tumor cells [reviewed in (9)]. The TCRs expressed by CD8 killer cells recognize pHLA complexes on tumor cells and are necessary and sufficient to mediate durable remissions across various types of cancer immunotherapies, including checkpoint inhibitors (CPIs), adoptive cell therapies and cancer vaccines (10–12). Combined, monoclonal antibodies (mAbs) and TCRs constitute the primary molecular mediators of the adaptive immune response across a large variety of disease indications. Given their critical role in mediating productive immune responses, mAb and TCRs are being pursued for therapeutic development of high potency modalities in Oncology and beyond. In this review, we focused on the applications of pHLA targeting compounds from various origins for the treatment of cancer.

One key feature distinguishing the interactions between antibodies and TCRs with their respective target antigens are their relative low target binding affinities. TCRs bind to their pHLA targets with apparent binding affinities ranging from 1 to 100 uM (13, 14), while most therapeutic antibodies display target antigen binding affinities in the nano- and sub-nanomolar ranges (15). The main driver for the 10^3^ - 10^6^-fold difference is that TCRs engage with their targets in higher order TCR-clustering, forming a highly controlled, multivalent receptor- target binding complex, with several hundred copies of a TCR bound to an equal number of their respective pHLA targets. Such TCR-pHLA interactions occur in spatially confined, 3 dimensional structures at the interface between T- and tumor cells, known as the immunological synapse. The formation of such multimeric target receptor complexes greatly increases the overall binding avidity and after reaching certain threshold levels, trigger TCR signaling and ultimately tumor cell killing.

Similarly, low affinity antibodies are also formed following an initial exposure to an antigen. However, antibody producing B cells, such as plasma cells, undergo a series of well conserved genetic events leading to affinity maturation of antibodies *via* somatic hypermutation, class switching and a clonal selection process resulting in continuously increasing antibody affinities. The impact of increasing the affinities and overall avidities of antibodies on their pharmacological properties have been studied intensively and were summarized recently (16). In the mAb context, the affinity between an antibody Fab fragment and the antigen is defined as zero-order avidity. Additional avidity increases are provided by the bivalency of Fab-antigen interaction (first-order), simultaneous Fab-antigen interactions and Fc-Fc or Fab-Fab interactions (second-order) and interactions between the mAb and effector cells (third order).

Natural immune responses are associated with the formation of polyclonal antibodies, varying in their isotypes, binding affinities, and epitopes, all contributing to increased binding avidity due to higher order avidity interactions. For therapeutic development, however, the use of monoclonal antibodies is the mainstay because of their simplified manufacturing requirements and streamlined regulatory paths, compared to lower affinity, multivalent antibodies, including IgM or IgA isotypes, which are more prone to aggregate formation during manufacturing, and thus have an increased risk for anti-drug antibody (ADA) induction and inflammatory reactions in clinical settings [reviewed in (17)]. To achieve meaningful pharmacological responses and to compensate for the lower overall avidity of monoclonal antibodies, there is a need to select for high affinity binders.

IgG1 based antibodies rely mostly on bivalent target antigen binding stoichiometries (1^st^ order avidity) and trigger a divers set of biological anti-tumor responses *via* their unique Fc gamma receptor (FcgR, third order) engagement. The responses include the activation of immune effector cells such as macrophages (Antigen dependent phagocytosis, ADCP), dendritic- and NK cells (Antigen dependent cellular toxicity, ADCC) among other mechanisms [reviewed in (18, 19)]. For efficient activation of NK cells, antibodies need to bind with high affinities to target antigens on the surface of tumor cells (20), thereby triggering NK cell activation and tumor cell lysis *via* engagement of FcyRs.

Ultimately, these differences in the biological, cellular, and molecular mechanism of action by which B- and T cells mediate tumor cell killing form the foundation for the different protein engineering approaches employed for targeting of pHLA complexes *via* TCRs or *via* the “antibody like” TCR mimic (TCRm) compounds, respectively. In particular TCRm compounds that engage with their pHLA targets *via* mono- or bivalent stoichiometry, depend strongly on higher target binding affinities to achieve anti-tumor activity, compared to TCRs, which bind to pHLA targets as multimeric complexes, requiring much lower target affinities to achieve pharmacological anti-tumor activities, due to much higher avidity effects.

## Structural, biochemical and physicochemical aspects of a productive TCR: pHLA interaction

The key features contributing to the exquisite target sensitivity and potency of low affinity, native TCRs towards pHLA targets on tumor cells are under intense investigation and have been reviewed previously (21). Importantly, TCRs can be activated by as few as 1-10 copies of a pHLA target presented on the surface of a tumor cell (22, 23). The main cause for such unique target copy number sensitivity of TCRs is the strong avidity effect generated by the multi-valent binding of several hundred pHLA complexes presented on tumor cells, with an equal number of TCRs expressed on activated T-cells, forming a “Velcro-like” high affinity binding interface forming the immunological synapse (24).

The potency of a T cell response following pHLA engagement of the TCR reflects the summary of multiple criteria, including affinity (25, 26) and confinement time of the TCR-pHLA complex (27) as well as structural aspects, including complex stability, docking geometry and conformational changes (28–31). In contrast to antibody-based therapeutics, the affinity of a TCR to its respective pHLA complex is poorly predictive for T cell activation and anti-tumor activities.

The formation of a catch or slip bond represents a collective property of the entire TCR-pMHC interface, whereby catch bonds prolong the stability and duration of the interaction, which is extended under force (32–34). The degree of catch bond formation was found to be most correlative with the productive signaling induced upon TCR-pHLA engagement and represents the result of structural chemistry combined with the energetic landscape of the TCR-pHLA interface (35).

In contrast to TCRs, TCR mimics engage with their targets mostly by first order avidity, and thus require much higher binding affinities and/or target densities to elicit comparable, apparent cellular binding affinities. As a consequence, high affinity TCRm compounds are needed to compensate for the absence of the avidity to achieve target specific antitumor activities with mono- or bivalent compounds at pharmacologically meaningful concentrations.

For the purpose of this review, we defined TCRm compounds as “any pHLA binding scaffold, including TCRs, IgGs and non-IgG scaffolds binding to a pHLA complexes with higher binding affinities compared to endogenous, native TCRs”, i.e. with a K_D_ of < 1uM.

Four main approaches are currently being pursued to optimize the anti-tumor activities of TCRm based therapies, as summarized below.

1. Affinity maturation to increase pHLA binding affinities towards nanomolar or picomolar ranges
2. Engineering of pharmacokinetic (PK) properties to increase therapeutic exposure levels in the circulation.
3. Introduction of additional potency enhancements to TCRms, either by increasing intrinsic effector functions *via* the redirection and activation of immune cells (mAbs and bispecifics) or by conjugation to cytotoxic payloads (ADCs).
4. Combining pHLA targeting with the highly sensitive TCR signaling machinery by using CAR constructs fused to a high affinity pHLA target binders (TCRm-CAR T or TCRm-CAR NK cells)

Antibodies or antibody-fragments are the most widely used modalities among biotherapeutics in oncology for targeting of cell surface antigens (36). The majority of the antibody-based TCRm platforms are designed with the intention to produce high affinity pHLA binders, and are based on *in vivo* immunization of laboratory animals with pHLA complexes or *in vitro* panning of libraries with yeast- or phage display technologies (37). In general, there is a strong correlation between increasing the pHLA binding affinity of TCRm compounds and their therapeutic potency when tested in *in vitro* pharmacology studies. In contrast, affinity maturation of full length TCRs in the context of cellular therapies, does not necessarily result in higher therapeutic potencies, because of additional physicochemical properties controlling TCR activation, such as the formation of catch bonds or proper docking geometry (30, 35, 38), as discussed above.

## Safety considerations when engineering TCRm- or TCR compounds

The first generation of both, TCRs and high-affinity, high-potency TCRm compounds came at the cost of novel cross-reactivities towards non-intended pHLA complexes, that were introduced during the affinity maturation process (39–41). Importantly, many of these engineered cross-reactivities could not be detected preclinically with the technologies available at the time. Due to significant species differences in MHC, TCR and target antigens, there is a paucity of relevant toxicology models to evaluate TCRm compounds, either *in vitro* or in laboratory animals. Consequently, some cross-reactivities that were introduced during affinity maturation of TCRs were first noticed during clinical testing, whichled to discontinuation of clinical trials at early stages during dose escalation (42, 43).

The standard biochemical specificity assays employed for TCRs and TCRm compounds include target selectivity studies using conventional alanine- or X-scanning of the target peptide. Unfortunately, these technologies are insufficient to identify the full spectrum of target peptides potentially recognized by affinity engineered TCR- or TCRm compounds (44). In particular, the class of peptides with limited sequence similarities with the on-target, tumor specific peptide, are difficult to detect when using these methods [reviewed in (6)].

Another main difference between cell surface antigens and pHLA targets is the difference in their overall diversity. For example, the number of theoretical peptides that can be presented on pHLAs in humans was estimated to exceed 11 million (45). When combined with splice variants, non-synonymous mutations and non-coding, retroviral integration sequences, the total number of theoretical targets can reach close to 10^15^ (46). This is in stark contrast to the number of total membrane surface antigens known in humans that are expressed on tumors (47). Therefore, additional technologies outside the conventional Ala- or X- scan methods are needed to cover the entire pHLA target space in humans, as both approaches have diversity limits of 10^3^ peptide sequences per TCR or TCRm screened [reviewed in (2, 5, 48)]. Based on the experience with the first-generation compounds, it is paramount to achieve highest levels of target selectivity while avoiding cross-reactivity with pHLA complexes containing non-relevant peptides at the preclinical stages of drug development, to decrease clinical attrition rates of TCRms.

In the following paragraph, we summarize the various technologies employed for the development of high affinity, pHLA targeting TCRm compounds and the most advanced therapeutic TCRm based programs currently developed in the clinic for the treatment of cancer, including TCRm-CD3 bispecific s, ADCs and CAR T and CAR NK cell therapies.

## Antibody based TCRm compounds for the development of redirected T cell therapeutics

Protein based, targeted immunotherapies (biotherapeutics) offer an “off-the-shelf” therapeutic intervention, differentiating them from the personalized approach of adoptive cell therapies (ACTs). TCRm compounds redirecting T cells toward the tumor, known as TCRm-CD3 bispecifics, combine the best of both modalities, adoptive cell therapies and biotherapeutics, as they integrate specific recognition of intracellular tumor antigens of cell therapies with the pharmacologically proven ability of CD3 engagers to recruit immune cells to the tumors, resulting in potent anti-tumor activities.

**Figure 2:**
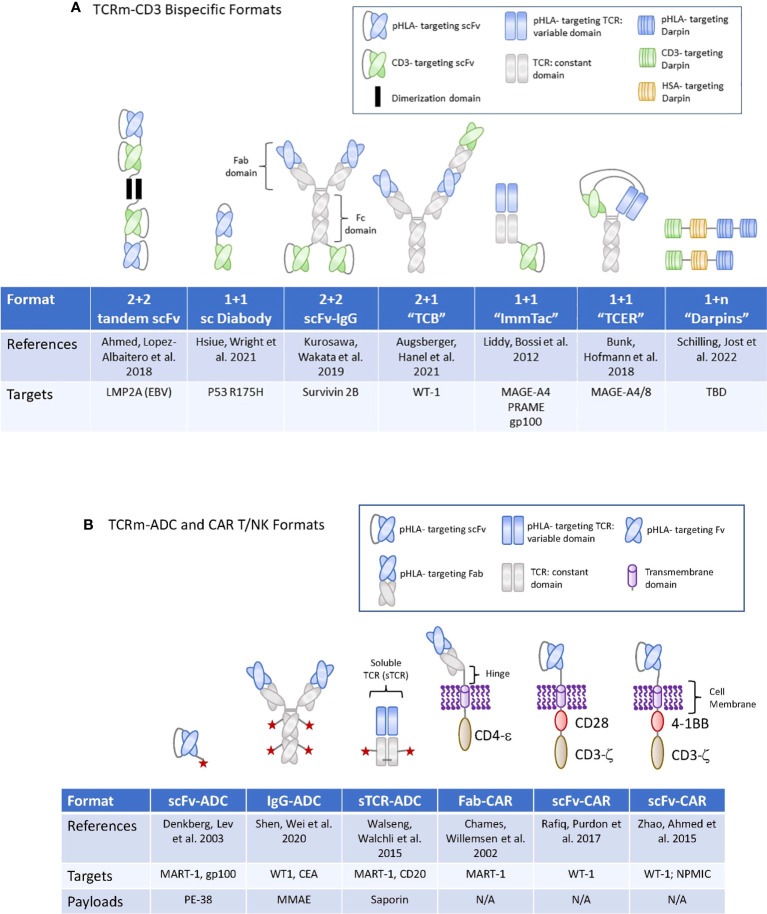
TCRm formats used for high potency modalities in Oncology. **(A)** (upper panel): Graphic display of the therapeutic formats used for TCRm-CD3 bispecific compounds. TBD, to be determined. **(B)** (lower panel): Graphic display of the therapeutic formats used for TCRm-ADCs and TCRm-CAR T and -CAR NK cells. N/A, not applicable.

For the development of IgG based TCRm compounds, highly diverse human antibody phage display libraries were screened for rapid selection of single-chain variable fragments (scFvs) or Fabs, capable of recognizing peptide/HLA complexes with sufficiently high affinities (49, 50). Several TCRm-CD3 bispecifics induced potent tumor cell killing when tested against human colorectal tumor cells grown *in vitro*, or eliminated transformed human B cells grown in mice (51, 52), (**Table 1** and **Figure 2A**). These first generation TCRm-CD3 bispecifics consisted of a single chain diabody fused to a CD3 binding scFv. When incubated with human PBMCs or T cells, they induced a polyclonal T cell response against cancer cells expressing the corresponding pHLA antigen. To ensure specificity of binding to the neoantigen peptides, selective binding towards peptides containing a p53 mutation (p53 R175H), but not the wild type p53 antigen peptide expressed on normal tissues (50), was reported. Importantly, anti-tumor activity was shown to be dependent on the formation of an immunological synapse between tumor- and T cells, identifying efficient immunological synapse formation as an important experimental biomarker to select for TCRm-CD3 bispecific compounds with highest anti-tumor activities.

**Table 1:** TCRm compounds developed as bispecifics in Oncology.

Modality	Sponsor	Target/HLA	Stage	Indication	Treatment	Trial ID/ref
CD3-Bispecific (TCR based)	Immunocore	MAGE-A4/A2	Phase 1/2	Solid tumors, prim and mets 2L	IMC-C103C; PD-L1 (Atezo)	NCT03973333
CD3-Bispecific (TCR based)	Immunocore	PRAME/A2	Phase 1/2	Solid tumors, prim and mets 2L	IMC-F106C; PD-L1	NCT04262466
CD3- Bispecific (TCR based)	Immunocore	GP100/A2	FDA approved for Uveal Melanoma in Jan, 2022 Phase 1/2 cutaneous melanoma	Uveal melanoma, cutaneous melanoma, other solid tumors	IMC-GP100/ Tebentafusp-tebn, Kimmtrak	NCT03070392 (53) (7)
CD3-Bispecific (TCR based)	Immunocore, Roche	MAGE-A4/A2	Phase 1	Solid tumors	IMC-C103C	NCT03973333 (54)
CD3-Bispecific (IgG based)	GSK	NY-ESO-1/A2	Phase ½	Synovial melanoma	GSK01	NCT01343043 (55)
CD3-Bispecific (TCB, IgG based)	Roche	WT-1/A2	Phase 1	Relapsed, refractory AML	RG6007	NCT04580121 (56)
CD3-Bispecific (TCB, IgG based)	Roche	MAGE-A4	Phase 1	Solid tumors	RG6129 RO7444973	NCT05129280
CD3-Bispecific (TCR based)	Immatics	MAGEA4/8/A2	Phase 1	recurrent and/or refractory solid tumors;	TCER IMA401	NCT05359445 (57)

A different therapeutic format was chosen for the development of a T-cell bispecific (TCBs), targeting the WT1 antigen, which is overexpressed in AML patients. The WT1 peptide is present in most cancer patients expressing HLA A*02. The pHLA TCB binders were selected from a synthetic human antibody phage display library (58) and integrated into a 2 + 1 format, wherein two pHLA binders are combined with one CD3 binder (56). The pHLA selectivity was assessed by conventional alanine scanning methods combined with a novel, peptide MHC array method, which revealed that six of the nine amino acids of the target peptide, were involved in the recognition by the IgG1 class antibody (**Table 1** and **Figure 2A**).

Additionally, human nonimmune phage display libraries were used to generate TCRm Fabs against a number of pHLA targets, including hTERT (59), MUC1 (60), and NY-ESO-01 (61). Although these compounds were not reformatted into bi-specific molecules for therapeutic evaluation, these studies provided evidence for specific binding of Fabs towards the pHLA complexes on cells expressing the target gene and HLA type of interest. Interestingly, these antibodies also served as blocking reagents when tested in the context of cytotoxic T cell responses towards the same antigens. Finally, the TCRm Fabs were shown to have additional utility as reagents to directly quantify the pHLA copy numbers on the surface of cells (59).

While some of these early studies with TCRm compounds provided evidence for potent pharmacological activities of Fabs in the context of TCRm-CD3 bispecifics , additional improvements in their pharmacological properties, including half-life extension, and reductions in their cross-reactivities are required to optimize their anti-tumor activities. For a summary of TCRm-CD3 bispecific compounds undergoing clinical development, please see **Table 1** and **Figure 2**. A for their molecular formats.

## TCRm compounds for the development of CART or -NK cells

Cellular therapies such as TCR-T or CAR T or -NK cells have several advantages over protein therapeutics [reviewed in (62)], in particular their potential to indirectly deliver a variety of immune-modulatory molecules like cytokines to the tumor microenvironment. However, despite the successes in the treatment of liquid tumors, their advancement towards solid tumors was hampered due to the lack of targets with sufficiently high tumor specific expression [Reviewed in (63)]. Therefore, the identification of antigens with improved tumor- to normal tissue expression ratios, including certain types of pHLA targets, is critical for the success of this therapeutic modality outside liquid tumor indications [reviewed in (6)].

For targeting of pHLAs *via* the CAR T approach, a non-immune Fab library was used to isolate a TCR-like Fabs against HLA A1 (MAGE-A1). Subsequent rounds of affinity maturation using a combination of light chain shuffling and heavy chain mutagenesis was employed to generate an anti-HLA-A1 (MAGE-A1) TCRm Fab, which was subsequently cloned into a CAR backbone. High- and low affinity Fabs binding to MAGE-A1 were fused to the FcεRIg chain signaling molecule and construct were transduced to T cells (**Figure 2B**). The resulting CAR T cells expressing the high-affinity chimeric receptor exerted higher lytic activity and faster kinetics (64). In addition, they required lower density of pHLA targets to secrete TNF-α after engaging with the target tumor cells and elicited the most potent tumor cell killing activity (65). These early studies provided proof of concept that pHLA specific Fabs have utility for tumor targeting in the context of CAR T cell therapies.

More recent reports suggested that targeting of pHLA complexes with a scFv fragment inserted into a CAR resulted in potent anti-tumor activities (66). This group utilized phage display technology to identify scFvs binding to the WT1/HLA-A*02:01 complex. The variable heavy and light chains were connected *via* a (Gly4Ser)3 spacer domain in conjunction with an immunoglobulin k-leader sequence. The CAR consisted of a CD28 transmembrane and cytoplasmic signaling domains along with the CD3ζ signaling domain (**Figure 2B**). Another group engineered WT1/HLA-A02:01 specific scFv in the context of a 4-1BB containing CAR and a scFv-Fc fusion antibody, with binding affinities of 3 nM and 2 pM, respectively (67) (**Figure 2B**). Such TCRm-CAR Ts displayed direct cytotoxic activity against WT1+ cancer cell lines *in vitro*, while the scFv fusion antibody inhibited tumor growth in animal models of cancer, in part through Fc mediated effector functions. In a separate study, WT1/HLA-A*02:01 specific scFvs were identified *via* phage display and reformatted into an IgG TCRm known as ESK1 (68). The ESK1 antibody was active against established acute lymphocytic leukemia in mouse models (15). When the ESK1 mAb was additionally formatted as a bispecific T-cell engager, in conjunction with a CD3 binder, it effectively redirected T cells to kill tumor cells *in vitro* (69).

Other studies employed a two-step procedure for the optimization of Fabs against peptide/HLA complexes. In the first step, high-resolution structures of two Fabs bound to HLA-A*0201/NYESO-1157–165 and the corresponding 1G4 TCR were solved and compared. In a follow-on step, the structural data was used to modify the antibody libraries based on the original Fab. The key amino acids of the Fab in contact with the central motif of the peptide were kept, and the remaining residues were randomized in positions where the side chains could be enabled to interact mostly with the peptide but not with the HLA backbone (70). The final Fab candidate achieved a 20-fold affinity improvement (2– 4 nM) through two amino acid substitutions in the light-chain and exceeded the affinity of the original TCR by 1,000-fold. The Fabs were grafted onto a TCR and following retrovial transduction of CD3^+^ T cells, moderate activity in target cell lysis of T2 cells expressing the NY-ESO-1 target peptide, was reported, with the higher affinity Fab construct inducing higher levels of IFN-γ release and cytotoxicity.

More recently, a yeast surface display library was used to isolate high affinity, scFvs specific for a neoantigens from an oncogenic driver gene nucleophosmin (NPM1c), which is present in about 35% of patients with acute myeloid lymphoma (AML). The yeast display library was generated using the full repertoire variable region gene fragments isolated from naïve, human splenic B cells. Yeast display allows for the selection of high affinity binders by increasing the pHLA binding stringency based on scFv binding to the target pHLA complex on the yeast cell surface by flow cytometry. The diversity of the scFv libraries screened can reach 1 x 10^7^ to 1 x 10^9^ different clones (71). The scFv binder was cloned into a chimeric antigen receptor (CAR) construct containing a CD8a hinge and transmembrane (TM) domain, a 4-1BB costimulatory domain, and a CD3ζ activation domain, followed by a self-cleavage P2A and GFP (**Figure 2B**). CAR T cells expressing the construct exhibited potent cytotoxicity against neo-antigen positive leukemia cells and primary AML blast, but not neoantigen negative control cells (72). Since both, 4-1BB and CD3ζ activation domains are naturally used in NK cells for signaling and activation, the same construct, when introduced to NK cells *via* lentiviral vectors and combined with a membrane bound form of IL-15, induced comparable or more potent cytotoxicity against NPM1c positive AML tumor cells when tested in preclinical models (73). Combined, these early studies demonstrated the feasibility of targeting low-copy number pHLA targets with conventional CAR T or -NK cells, resulting in eradication of tumor cell lines expressing the endogenous pHLA target in preclinical mouse tumor models. For a summary of TCRm based CART programs currently being developed in the clinic, please see **Table 2** and **Figure 2B** for their molecular formats.

**Table 2:** TCRm compounds developed as CAR-T or -NK cells in Oncology.

WT-1-CAR T	Eureka Therapeutics	WT-1/A2	Preclin	Leukemia and ovarian cancer		(66–68)
AFP-CAR T	Eureka Therapeutics	AFP/A2	Phase1/2	Hepatocellular	ET140203 cells	NCT03349255 (74)
NPM1c-CAR T and CAR NK	MIT, Cambridge	NPM1c/A2 (neoantigen)	Preclin	Leukemia and AML		(72, 73)

## TCRm compounds for the development of ADCs

There has been a strong focus to adapt TCRms for bispecific therapies due to the unmatched potency of immune effector cells to induce tumor cell killing following their recruitment and activation in the tumor (75). In contrast, there are fewer reports on the development of TCRm based ADCs, partly due to initial concerns that the pHLAcopy numbers on tumor cells, which range fom10 to 5x 10^3^, are below the threshold number required for conventional cell surface antigens to internalize sufficient amounts of cytotoxic payloads to induce tumor cell death (76).

The most successful ADC payload class in the clinic are tubulin inhibitors, including vinca alkaloids (auristatins) and maytansine payloads, both of which rely on medium to high expression levels of cell surface antigens in order to induce meaningful anti-tumor responses. Tubulin inhibitors provide an additional level of tumor specificity, as their mechanism of action is cell cycle dependent. Since most normal tissue cells do not undergo active cell division at the rate tumor cells do, low levels of normal tissue expression of the target antigen is tolerated. Conjugates with the more potent, non-cell cycle dependent, DNA targeting class of payloads, including DNA alkylators and crosslinking compounds such as pyrrolobenzodiazepines (PBDs), cyclopropapyrroloindoles (CPIs), duocarmicins, calicheamicin and others were shown to induce regression of tumors expressing the target antigen at low copy numbers [reviewed in (77)]. However, such DNA targeting payloads render the ADC less tolerant towards target antigens expressed on normal tissues. Consequently, DNA targeting ADCs were most successfully developed for the treatment of liquid tumors, as the toxicity resulting from ablation of normal lymphocytes expressing the target antigen is better tolerated compared to targets expressed on normal epithelial cells. Targeting of pHLA subclasses expressed at higher tumor-to normal tissue ratios compared to the current cell surface antigens, may potentially enable the expansion of the utility of high potency linker payload classes toward solid tumor indications.

The earliest reports documenting anti-tumor efficacy with ADCs targeting pHLA targets were published about 20 years ago (78, 79). For these constructs, a phage display library was used to select for human Fabs binding to the pHLA-A2 complex. In these pioneering studies, anti-tumor activity was demonstrated with a conjugate consisting of a Fab targeting MART-1 or gp100 fused to the bacterial toxin *Pseudomonas* exotoxin A (78, 79) (**Figure 2B**). Follow on studies used the ribosomal inhibitor saporin conjugated to soluble TCR (sTCR) targeting a peptide withing MART-1 (MART-1p/HLA-A*02:01) or a peptide within CD20 (CD20p/HLA-A*02:01), both in the context of HLA-A2 (80). For these experiments, an expression construct encoding for the TCRα and -β chains of the TCR (αβTCR). devoid of the transmembrane and intracellular domains, was used, linked by a ribosomal skipping 2A sequence to enable equimolar production of the chains (**Figure 2B**). The saporin toxin conjugated to streptavidin was bound to the sTCR and selective internalization was demonstrated to occur in a target antigen specific manner. Combined, these early studies provided evidence for the potential of ADCs targeting pHLA complexes to internalize rapidly, leading to accumulation of sufficient amounts of intracellular payloads to induce tumor cell death. When tested *in vivo*, some conjugates induced anti- tumor responses at pharmacologically relevant exposure levels. However, while these initial TCRm-immunotoxin compounds induced meaningful anti-tumor responses against high copy number pHLA targets, they were insufficient against tumors expressing less than 10,000 peptide/HLA copies per cell, therefore limiting the utility towards targeting of a few, most highly expressed pHLA targets.

More recently, additional proof of concept data was generated with TCRm based ADC compounds consisting of a scFv recognizing WT1 or CEA pHLA complexes (81, 82). When conjugated to high potency payloads, including the DNA alkylating agent Duocarmycin (83), conjugates induced strong anti-tumor responses *in vitro* and *in vivo* against tumor cells expressing endogenous levels of pHLA targets, including breast and colorectal tumor cells, which present the respective peptide/HLA targets at physiological levels of 350–2,000 copies per cell. These findings suggested that ADCs conjugated to the more potent, non-cell-cycle dependent payloads including DNA alkylating agents, were sufficiently potent against pHLA targets expressed at endogenous levels of 5000 copies/per cell or less. A summary of TCRm-ADCs developed preclinically can be found in **Table 3** and their molecular composition is summarized in **Figure 2B**.

**Table 3:** TCRm compounds developed as ADCs in Oncology.

TCRm-ADC (vcMMAE, IgG based)	Huabo Biopharma	KRAS-G12V/A2	Preclin	Pancreatic, colon and lung cancer, melanoma	2E8-MMAE And 2A5-MMAE,	(82)
TCRm-ADC (vcMMAE, IgG based)	Huabo Biopharm	WT1/A2	Preclin	Kidney, urinary, bladder	ESK-MMAE, and Q2L-MMAE	(81)
TCRm-ADC (Saporin, Duocarmycin, IgG based)	University of Texas	CEA/A2	Preclin	Breast, CRC	FLS- and YLK- ADCs	(83)

Despite the progress made with ADCs targeting pHLAs, additional work will be needed to identify linkers with optimal intracellular payload release properties. The current crop of linkers was selected based on intracellular trafficking pathways engaged by cell surface antigens, which predominantly cycle through endosomal- and lysosomal compartments (84, 85). In contrast, pHLA complexes internalize *via* the endocytic recycling pathways of MHC molecules (86). The circumstance that both internaliztion pathways engaged by pHLAs and cell surface antigens include early- and late endosomal-, as well as lysosomal compartments (87) may explain the anti-tumor activity observed with current linker payload technologies developed for conventional cell surface antigens. However, there are unique intracellular trafficking compartments involved in pHLA trafficking, such as recycling through the ER compartment, which warrants a careful evaluation of the current ADC linker technologies and provides an opportunity to further optimize linkers for payload release of ADCs targeting pHLAs.

## TCR derived, high affinity TCRms for the development of bispecifics

The canonical interaction between a TCR and the corresponding pHLA target complex positions the TCR diagonally across the HLA-binding groove, placing the CDR3 loops centrally over the presented peptide and the CDR1/2 loops placed primarily over the HLA helices. These features enable endogenous TCRs to detect the pHLA complex in a peptide dependent manner [reviewed in (88)]. Because of the unique steric requirements and architecture posed by the TCR-pHLA interface, engineering of the TCR scaffold may provide an advantage over other scaffolds. The success of this approach depends on overcoming several barriers, such as the relative low binding affinity between naturally selected TCRs and their targets.

To effectively engineer and affinity mature TCRs, conventional site directed mutagenesis technologies were employed with a focus on changing the residues within TCR domains that directly interact with the target pHLA complex. The majority of CD8+ T cells recognize their peptide antigens on HLA class I molecules *via* the αβTCR. Each of the TCR chains contains three complementarity-determining regions (CDRs) which are generated by V(D)J recombination, forming a total of six flexible loops, known as complementary determining regions (CDRs) that contact HLA class I and the presented peptide (89). In general, the HLA class I heavy chain is in contact with the germline-encoded CDR1 and CDR2 domains, while the hyper-variable CDR3 is in direct contact with the HLA bound peptide (90). Most affinity matured TCRs were generated by using phage or yeast display libraries combined with targeted mutagenesis of the antigen binding residues in the CDR-1, 2 and 3 loops. These libraries were subsequently selected for binding to soluble pHLA molecules under conditions of increasing stringency to identify high affinity TCR mutants that bind selectively to the pHLA target of interest. More recently, transgenic mice were generated that express human TCRs in the context of the corresponding human HLA molecules. These engineered mice allow for the generation of a large variety of human αβTCRs towards selected targets (91).

The TCR alpha and beta chains are less stable when expressed as soluble proteins, compared to antibodies, and thus require additional stabilization which is commonly achieved by engineered interchain disulfide bonds or interdomain stabilizing mutations (83, 92); reviewed in (93). Several soluble TCR based bispecifics with improved stabilities and with low nano- to picomolar binding affinities to pHLA complexes are currently undergoing clinical testing [**Table 1** and (8, 94, 95)].

TCR affinity maturation was employed in the context of bi-specific T cell engagers known as ImmTACs. ImmTACs are composed of an anti-CD3 scFv linked to an engineered full length extracellular domain of an antigen specific TCR. These TCRs have been stabilized and affinity matured resulting in sub-nanomolar affinities and binding to low copy number pHLA targets. ImmTACs targeting the pHLA target GP100 have demonstrated efficacy in the clinic when targeting uveal melanomas (53). Additional programs using this format targeting NY-ESO-1, MAGE-A4 and PRAME are currently being developed (83).

A similar TCR affinity maturation approach was applied to generate bispecific T Cell Engaging Receptor (TCER) compounds as CD3 bispecifics. For this platform, an *in vitro* screen was employed to identify multiple natural TCRs binding to a select pHLA target expressed on artificial antigen-presenting cells, followed by affinity maturation of the variable α- and β-TCR domains and concomitant selection for highest target specificity. The mature single-chain TCRs (scTv) are then cloned to the variable domain of the light and heavy chain of an IgG1 mAb binding to CD3. This diabody based, bispecifc format is grafted onto an effector function-silenced IgG1 Fc domain, that contains additional knob and whole mutation to stabilize the molecule. TCER compounds display picomolar cellular potencies and greatly extended half lives compare to the non-half live extended ImmTac compounds (57). The molecular formats of TCRm-CD3 bispecifics composed of soluble TCRs are displayed in **Figure 2A**.

As shown in **Table 1**, a total of eight TCRm-CD3 bispecific compounds with different formats are currently being developed in the clinic, four of which are targeting a similar pHLA target (MAGE-A4). The clinical data generated will help to better understand the contributions of target selectivity, exposure levels, pHLA targeting format to clinical response rates and inform future generation of TCRm based compounds in Oncology.

## Alternative protein scaffolds for TCRm development

When scaling production for manufacturing, antibodies can display variabilities in their expression yields, in their tendencies to form aggregates and in their reliance on disulfide bonds for stability and to maintain appropriate glycosylation, and in most cases, require a mammalian cell expression system. These circumstances triggered a search for alternative protein scaffolds to address these manufacturing liabilities while achieving the same target binding affinity and specificity as antibodies. Previous attempts to generate non-antibody-based binders relied on loop or surface randomization of different protein scaffolds, which are fixed in dimension (96, 97). Some of the early scaffolds were based on protein A, fibronectin, lipocalins or green fluorescent protein and achieved nanomolar target binding affinities along with high specificities [reviewed in (36)]. Alternative approaches focused on repeat proteins characterized by a series of homologous structural repeats, which stack against each other to form an extended protein domain with a continuous hydrophobic cores (98). This architecture allows for the generation of bindings specificities not only based on mutations but also by insertion, deletion or shuffling of repeats (99). Ankyrin repeat (AR) proteins can mediate protein-protein interactions in a variety of environments (100), with reported binding affinities that can reach low nanomolar (101, 102). The first-generation AR proteins consist of stacked, 33 amino acid repeats, each forming a β-turn followed by two antiparallel α-helices are known as Darpin class protein binders. Each unit is followed by a loop reaching the β-turn of the next repeat (103). Combinatorial libraries consisting of consensus-darpin sequences of varying numbers with randomized potential interaction surfaces have been generated and used successfully to generate binders to a variety of targets, including pHLA complexes. The elements of the libraries were well expressed, soluble, thermodynamically stable and displayed the typical AR domain fold (104). The high expression level of designed AR proteins combined with their high thermo-dynamic stability (105), the absence of cysteines, the rapid enrichment of binders and their low nanomolar affinities may render them an attractive alternative to antibody scaffolds for pHLA targeting. The molecular formats of TCRm-CD3 bispecifics composed of Darpins are displayed in **Figure 2A**.

## Challenges in the development of TCRm compounds

### Optimizing target specificity of pHLA binders

One of the key challenges when targeting pHLA complexes is the large number of potential targets that can be recognized by TCR based therapeutics. Therefore, comprehensive profiling of TCR and TCRm compounds is required to achieve high levels of target saturation and specificity. This has become even more relevant after recent reports suggested that the intended target peptides of TCRs or TCRm compounds often differ in only one or two amino acids from unintended, off-target pHLAs that can be present on healthy tissues [reviewed in (5)].

More recently, several high throughput peptide panning methods were described to more comprehensively profile TCR or TCRm compounds for target selectivity during the engineering process. These technologies carry the potential of eliminating off-target binding introduced during the affinity maturation process [**Figure 1**, reviewed in (5)].

In addition, pHLA targets represent a challenge for protein engineering, as only a small area of the binding interface between the pHLA complex is accessible to the binders. In the case of antibodies binding to the WT-1 pHLA complex for example, only between 12% and 27% of the pHLA surface area is directly involved in binding to the IgG binder (56). An optimal steric fit between binders and the pHLA target complex is therefore critical to achieve highest levels of target specificity and affinity. The choice of the protein scaffold providing optimal rigidity characteristics and sufficiently large binding interfaces with the pHLA target complex is critical for success of this approach, to minimize binding of non-intended, off-target peptides. It remains to be determined which of the protein scaffolds discussed above are best suited to provide the highest target peptide specificity to prevent cross reactivities towards other, unintended, non- tumor pHLA targets.

### Overcoming treatment resistance towards TCRm compounds

The mechanisms underlying the development of resistance towards treatment with pHLA targeting compounds was studies in various translational studies in the clinic. Several studies conducted with TCR-T cells targeting pHLA targets suggested that pHLA downregulation, as a consequence of mutations acquired during treatment, either reduced pHLA presentation or decreased IFN-y production in tumor cells (106, 107). Such pHLA downregulation is most prominently associated with the development of resistance towards immunotherapy, including CPIs. Clinical studies conducted with CPI compounds identified mutations in the pathways involved in proteolytic processing, HLA alleles (108, 109), peptide loading (110), intracellular trafficking of pHLA complexes between the ER and the cell membrane (111) and cell surface presentation which all contributed to reduced overall pHLA presentation on tumor cells [reviewed in (112].

Some early reports studied the genetic changes associated with the development of resistance towards treatment with TCR-T cells, when administered to melanoma cancer patients with multiple metastatic lesions (113). The data corroborates with other studies in identifying the key mediators of CPI responses to be CD8 effector T cells in the tumors [reviewed in (107)] and pHLA downregulation as main path for tumor escape. In conclusion, there are multiple independent lines of research suggesting that mutations within elements of the pathways associated with antigen processing, peptide loading and intracellular trafficking of pHLA complexes in tumor cells correlate negatively with response to treatment to immune-oncology compounds [reviewed in (107, 112)].

To overcome the development of resistance towards TCR based therapies, several approaches are currently being pursued, as outlined below. A common strategy to address pHLA target downregulation is concomitant targeting of multiple pHLA targets that are co-expressed on the same tumor. In this context, multi target binder formats that can accommodate multiple pHLA targets are worth further exploration (113). Alternatively, concomitant targeting of class I and class II targets may avoid the development of resistance towards one target class, because different molecular pathways are involved in class I and class II peptide loading and presentation. In support of this concept, a recent study identified a subset of cytotoxic CD4^+^ T cells in bladder tumors treated with anti-PD1 immunotherapy, which exerted killing of autologous tumors *ex vivo* (114). Finally, combination treatments with compounds leading to upregulation of pHLA complexes on tumor cells are being pursued. Among the stimulator of pHLA target expression are epigenetic drugs and IFN-α (115). Both enhance tumor antigen expression by upregulation of gene involved in lysosome, phagosome, and antigen processing and presentation pathways (116) when tested in preclinical tumor models.

In conclusion, the lessons from these preclinical and clinical studies investigating the development of resistance towards TCR based therapies have great potential to guide the future clinical and translational development of TCRm based therapies targeting solid tumors

## Author contributions

All authors listed have made a substantial, direct, and intellectual contribution to the work and approved it for publication.

## Acknowledgments

We would like to thank Leah V. Sibener and Alejandro Ramirez for critical reading of the manuscript and their helpful comments and discussions.

## Conflict of interest

The authors declare that the research was conducted in the absence of any commercial or financial relationships that could be construed as a potential conflict of interest.

## Publisher’s note

All claims expressed in this article are solely those of the authors and do not necessarily represent those of their affiliated organizations, or those of the publisher, the editors and the reviewers. Any product that may be evaluated in this article, or claim that may be made by its manufacturer, is not guaranteed or endorsed by the publisher.
